# Tropical fruit-derived *Lactiplantibacillus* as potential probiotic and antifungal agents against *Fusarium oxysporum*

**DOI:** 10.1038/s41598-025-85190-0

**Published:** 2025-01-16

**Authors:** R. Vasundaradevi, M. Sarvajith, S. Divyashree, N. Deepa, Premila N. Achar, M. Y. Sreenivasa

**Affiliations:** 1 Molecular Mycotoxicology Lab, Department of Studies in Microbiology, University of Mysore, Manasagangotri, Mysuru, 570 006 India; 2 Department of Molecular and Cellular Biology, Kennesaw State University, Kennesaw, GA30144 USA; 3 Present Address: WDRC, Biological and Environmental Science and Engineering Division, King Abdullah University of Science and Technology, Thuwal, 23955-6900 Kingdom of Saudi Arabia

**Keywords:** Microbiology, Antifungal agents

## Abstract

Fifty-five lactic acid bacteria (LAB) were isolated from seven selected tropical fruits, with *Solanum nigrum* exhibiting the highest LAB prevalence and *Couroupita guianenis* and *Musa* fruits showing the lowest counts. Two strains isolated from *Ficus racemosa* demonstrated significant antifungal activity against *Fusarium oxysporum*. 16S rDNA sequencing identified these strains as *Lactiplantibacillus plantarum* MYSVCF3 and *Lpb. argentoratensis* MYSVCF5. The isolates displayed adaptability to a broad range of environmental conditions, including temperatures of 10–45 °C, pH 2–6, and salt up to 7%. The strains tolerated simulated gastrointestinal conditions of acid (pH-2), phenol (0.6%), and bile (0.3%) suggesting potential probiotic attributes. *Lpb. argentoratensis* MYSVCF5 inhibited *F. oxysporum*, two ESKAPE group bacteria (*P. aeruginosa*, *S. aureus*) plus *S. paratyphi* and *E. coli*. The cell-free supernatant (CFS) of *Lpb. argentoratensis* MYSVCF5 reduced the growth of fungal biomass by 94% and completely inhibited conidial germination, retaining activity even after extended cold storage. LC–MS/MS analysis identified organic acids in the CFS, with citric acid as the most abundant at 34.9 (± 0.3) µg/mL, followed by lactic (8.3 µg/mL) and malic acids (5.2 µg/mL). This study isolated a novel LAB, a potential candidate having probiotics and antifungal properties for application in food and agriculture.

**Supplementary Information:**

The online version contains supplementary material available at 10.1038/s41598-025-85190-0.

## Introduction

The contamination of food and agricultural products by *Fusarium oxysporum* poses a formidable challenge, resulting in substantial economic losses, food wastage, and compromised food safety^1,2^. *F. oxysporum* is widely distributed in the environment and is particularly endemic to tropical and subtropical areas where high temperatures and humidity favor its proliferation. Its resilience, due to chlamydospore formation, allows prolonged survival in soil, complicating control measures and leading to significant economic losses and food security challenges. Over 120 different plants are listed as hosts for *F. oxysporum*, causing various devastating plant diseases, including vascular wilt, sweet potato wilt, tomato wilt, and Panama disease^3−5^. In efforts to mitigate such losses, antifungal agents, including fumigants are commonly used for disinfection^6^. However, the effectiveness of these treatments is often inadequate and their usage is limited due to environmental consequences^3^. Alternatively, biological control strategies, including LAB-based interventions such as live microbes, cell-free supernatants, and purified compounds, offer a sustainable alternative to chemical methods, which are often ineffective against this pathogen’s persistence ^7^.

The endophytic microorganisms have the potential to produce secondary metabolites that can inhibit the growth of pathogenic fungi. The need for chemical fungicides can be reduced by harnessing natural capabilities while promoting sustainable agricultural practices^8^. Among these include lactic acid bacteria (LAB), extensively studied and recognized as probiotics due to their numerous health benefits^9,10^. Traditionally, these bacteria were isolated from fermented foods such as yogurt and consumed as probiotics^11,12^. Microbiologically, fruits are safer than other raw foods such as milk, meat, etc. Fruits due to their distinct chemical composition, buffering capacity, and presence of antagonistic compounds create a conducive habitat for bacterial adaptation, including probiotic strains like LAB^13^. Therefore, fruits serve as continuous and viable sources of probiotic bacteria. Moreover, with the growing demand for non-dairy alternatives, fruit offers a practical choice for populations with lactose intolerance, dyslipidemia, and those adhering to vegan lifestyle^14–16^.

Previous studies have successfully isolated LABs from a variety of fruits such as pineapples, oranges, watermelons, papaya, berries, etc^14,17^. *Lactobacillus rossiae, L. brevis, Weissella cibaria, Weissella paramesenteroides, Leuconostoc mesenteroides* and *Pediococcus pentosaceus* have been frequently identified as LABs in fruits^17,18^. However, most investigations have only focused on the LAB’s presence in fruits or its role in fermentation processes^18,19^.

Given that *F. oxysporum* is native to tropical and subtropical regions, we hypothesize that the LAB derived from tropical fruits possesses antimicrobial properties specifically, antifungal activities that are effective against *F. oxysporum*. LAB isolates from tropical fruits were chosen for their unique microbial diversity, shaped by high-acidity and humid conditions similar to those in which *F. oxysporum* thrives. These LAB strains are naturally adapted to these environments. By harnessing the antibacterial and antifungal properties of LAB derived from tropical fruits, we could develop natural, probiotic-based solutions to combat *F. oxysporum* infection. Not only would this be a safer option for human consumption, but it would also positively impact the environment by reducing the need for chemical fungicides. With this foreground, this study aimed to explore the antifungal potential of LAB from tropical fruits to develop environmentally sustainable and probiotic-based solutions for managing *F. oxysporum*. Specifically, the objectives were to (1) isolate LAB from selected tropical fruits, (2) evaluate the antifungal activity of these isolates against *F. oxysporum*, and (3) identify the active antifungal components. Seven tropical fruits (See supplementary Table S1) were selected for LAB isolation and subsequent antifungal screening.

## Materials and methods

### Isolation and preliminary characterization of LAB

LAB was isolated from assorted tropical fruits (Table S1) collected in and around the districts of Mysuru and Mandya (the state of Karnataka, India). LAB was isolated by enrichment technique in de Man, Rogosa, and Sharpe (MRS)^20^. All isolates were identified primarily based on morphological and biochemical characteristics: Gram stain, and negative catalase reaction (in 3% v/v H_2_O_2_).

### Screening of LAB isolates for antifungal activity

**Fig. 1:**
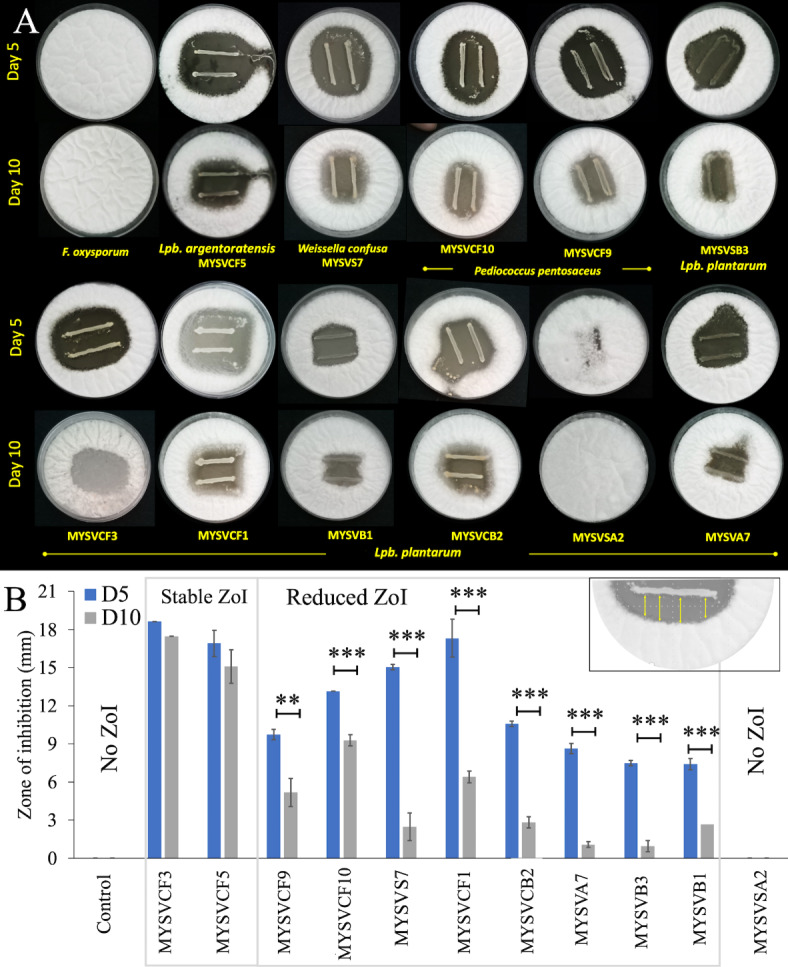
Growth inhibition of *Fusarium oxysporum* by LAB (**A**). Days 5 and 10 show the days after incubation. The zone of inhibition (ZoI) was segregated based on the statistical significance between days 5 and 10 (**B**). Inset (**B**) depicts ZoI measurement highlighted in yellow arrow between LAB and *F. oxysporum*. The X-axis in panel B shows strain identity. *p ≤ 0.05; **p ≤ 0.01; ***p ≤ 0.001.

The antifungal activity was assessed using the agar-overlay method^21^. LAB strains grown overnight were streaked onto MRS agar plates and incubated at 37 °C for 24 h. Molten-cooled PDA (0.7% agar) containing 10^6^ spores per mL was then gently poured onto the agar plates. Spore inoculum of *F. oxysporum* was prepared from potato dextrose agar (PDA) using 0.1 M PBS solution supplemented 0.2% Cap T, Tween 80. After solidification, plates were incubated at room temperature for 5 days to observe any inhibition zones around LAB colonies. The Zone of Inhibition (ZoI) was measured as the distance, in millimetres, between the LAB and *F. oxysporum* (Fig. 1B inset). Additionally, the stability of the ZoI was quantified by measuring the reduction in the ZoI due to the growth of *F. oxysporum* after 10 days of incubation at room temperature (Fig. 1B).

### Molecular identification of LAB with antifungal activity

Selected LAB strains were identified by sequencing their 16S rDNA. Overnight cultures in MRS broth were centrifuged and washed with PBS (0.1 M, pH 7.4). Genomic DNA was extracted using the GeNei™ kit following manufacturer’s recommendations. The 16S rRNA region was amplified using universal primers 8F and 1391R^9^. Amplicons were sequenced, and sequences were matched using the BLAST tool of NCBI. A phylogenetic tree was constructed using MEGA11 software^22^.

### In vitro characterization of selected LABs for probiotic attributes

The overnight grown isolates on MRS broth at 37 °C for 24 h were harvested by centrifugation at 8000 rpm for 10 min and used in the following assays. After incubation at the desired conditions, the cells were serially diluted and plated on MRSA. The bacterial viability was determined as CFU/mL^23^. LABs grown solely on MRS broth at 37 °C for 24 h were considered control. The results were then compared with those obtained from LABs exposed to stress conditions, including phenol, acid, and bile, to assess their tolerance and overall probiotic potential.

#### Tolerance to low pH, bile salts, and phenol

For the tolerance test at low pH, the cell pellet was resuspended in 5 mL of pH-adjusted MRS broth. The pH was adjusted to pH 2, pH 3, and pH 4 using 1 N HCl^24^. For the bile salt and phenol tolerance tests, the cell pellet was resuspended in MRS broth containing ox gall (0.3% (w/v)) or phenol (0.4 and 0.6% (v/v)) respectively. The isolates were incubated at 37 °C up to 4 and 24–48 h, respectively.

#### Growth at different temperatures and NaCl concentrations

100 µL of active cell culture was inoculated into 5 mL sterile MRS broth containing NaCl (3, 5, and 7% (w/v)). To evaluate the growth at different temperatures, 100 µL of active cell culture was inoculated into sterile MRS broth and incubated at 4, 10, 30, 37 and 45 °C. The cultures were incubated for 24–48 h before determining the viability.

#### Auto-aggregation and hydrophobicity index

For auto-aggregation, overnight-grown cell culture in PBS was monitored for absorbance at 600 nm at regular intervals to calculate aggregation (%) using the formula^25^: [(A_t _ − A_0_)/A_t_) × 100]. For cell surface hydrophobicity, bacterial suspension in xylene was assessed for absorbance at 600 nm, with hydrophobicity (%) computed as [(1 − A_t_/A_0_) × 100]. where A_t_ and A_0_ represent absorbance at 600 nm at any given time interval and at time 0 h.

### Antibacterial activity

LAB isolates and their cell-free supernatants (CFS) were tested against indicator microorganisms: *Escherichia coli* (ATCC 25922), *Salmonella paratyphi* (ATCC 9150), and two ESKAPE group pathogens: *Pseudomonas aeruginosa* (ATCC 15422), *Staphylococcus aureus* (ATCC 6538). The crude CFS was obtained by centrifuging 48-h-old LAB suspension at 8000 rpm for 10 min. The obtained supernatant was filtered using a 0.2-micron pore syringe filter (Millipore). A portion of the CFS was neutralized to pH 7 (n-CFS) using 1N NaOH to eliminate the effect of acidic component^26^.

### Agar-well diffusion using CFS of LAB

Indicator organisms grown overnight in nutrient broth were spread-plated onto nutrient agar plates. Wells of 6 mm diameter were aseptically made on the agar plates using a cork-borer, and 50 µL of CFS or nCFS was loaded. After incubation at 37 °C for 24–48 h, clear zone formation around the wells was examined, and antagonistic activity was calculated as the zone of inhibition.

### Safety profiling

#### Hemolytic activity

Hemolytic activity was assessed by plating overnight-grown LAB isolates on blood-agar plates^20^. Hemolysis was classified as β (complete), α (partial), or γ (none) based on zone characteristics. *Staphylococcus aureus* (ATCC 6538) served as the positive hemolysis reference strain^20^.

#### Antibiotic susceptibility

The susceptibility of LAB to common antibiotics was assessed using the disc diffusion method^27^ (Table 2). After incubation, the zone of inhibition was calculated as described and classified for potential *Lactobacillus* according to Charteris et al.^27^.

#### Antifungal activity by co-inoculation assay

Erlenmeyer flasks containing 50 mL sterile MRS were inoculated with 100 µL of LAB (~ 10^6^ CFU/mL) and 100 µL of *F. oxysporum* (10^6^ spores/mL)^20^ and incubated for 3–14 days at room temperature. Mycelial biomass was harvested on 3, 7, 10, and 14th day by filtration and dried at 80 °C for 2 h. The cell viability was determined as detailed above.

#### Antifungal activity using CFS of LAB strain

Microdilution method. To a sterile 24-well microtiter plate, 1.9 mL/well of CFS in various ratios (v/v) was prepared by mixing CFS directly with PDB. The extract concentrations of 0.3, 0.6, 0.9, and 1.2 mg/mL (w/v) were used. Each well received 100 µL of conidial suspension (10^6^ spores/mL). Controls included CFS or spores alone. After incubation at 30 °C, absorbance was measured at 600 nm. Minimum fungicidal concentration was determined as the minimum concentration of crude CFS extract needed to inhibit *F. oxysporum* growth.

#### Biomass inhibition

Erlenmeyer flasks containing 50 mL of crude CFS at varying ratios (5–25%, v/v) were prepared by mixing CFS directly with PDB. *F. oxysporum* fungal discs (7.5 mm diameter) were added, and flasks were incubated at room temperature. Control flasks contained only PDB. After 10 days, fungal mats were harvested, and dry weights were compared between CFS-treated and control flasks^20^.

#### Conidial germination inhibition

To a 24-well microtiter plate, each well contained a mixture of 100 µL of LAB (~ 10^6^ CFU/mL) and 100 µL of *F. oxysporum* (10^6^ spores/mL) in 1 mL of 0.1 M PBS, pH 7.4. For CFS assays, 10% CFS (v/v) and 100 µL of *F. oxysporum* (10^6^ spores/mL) were combined to the final volume of 1 mL in PBS (0.1 M, pH 7.4). Control wells contained only spores in PBS (0.1 M, pH 7.4). Plates were incubated at 30 °C for 24–48 h to evaluate conidial germination. The conidial germination was evaluated using a hemocytometer and expressed as percentage germination.

### Stability and activity of CFS of LAB strain

The CFS was treated with proteinase K (1 mg/mL), pH neutralization from pH 4.2 to pH 7 using 1 N NaOH, and high-temperature treatments by heating 100 °C for 15 min. The residual antifungal activity was assessed following the microdilution method^28^. Additionally, a shelf-life test assessed stability and inhibitory ability following > 8 months of freezing at − 20 °C and 4 °C, followed by antifungal experiments using the stored CFS. For this, to a 24-well plate, stored CFS in concentrations of 0, 10, 20 and 30% and 4 mm active *F. oxysporum* disk was incubated at room temperature.

### Identification and quantification of organic acids

The organic acid content of CFS was quantified by Liquid chromatography with tandem mass spectrometry (LC–MS) using UPLC BEH-amide (Waters) column using 10 mM ammonium acetate and acetonitrile (1:1), pH 8.5 as mobile phase. Complete details can be found elsewhere^29^.

### Statistical analysis

The data in the graph represents mean ± SD (standard deviation) derived from at least three replicates (n = 3). The effect of LAB on *F. oxysporum* was determined using the *t-test* in Microsoft Excel 2019 (Microsoft Corporation) software. The statistical significance was determined at *p* ≤ 0.05.

## Results

### Isolation of LAB

Seven tropical fruits were sampled during a dry season characterised with an average temperature of 30 °C and humidity exceeding 50% (see Supplementary Table S1). LAB counts ranged from 9–10 log CFU/g of fruit across the fruits, with *Solanum nigrum* and *Annona muricata* showing the highest counts at 10 log CFU/g of fruit each, followed by *Tinospora cordiofolia*, *Ficus benghalensis, Couroupita guianenis*, and *Musa*, each with 9 log CFU/g of fruit. Despite these high counts, only a subset of these colonies exhibited distinct pin-point and morphologically different colonies. *Solanum nigrum* had the highest count at 14 CFU, followed by *Ficus racemosa* at 10 CFU. The least number of colonies were observed in fruits *Courorpita guianenis* and *Musa* at 4 each (see Supplementary Table S1). Interestingly, the morphological distribution of LAB varied among the fruits. *Solanum nigrum, Musa*, and *Annona muricata* predominantly harbored rod-shaped cells, while *Ficus racemose* exhibited mostly cocci-shaped cells. Among the rods, *Annona muricata* had short-rod cell types, while *Tinospora cordiofolia* contained a uniform combination of cell types, including rods, short rods, and cocci.

### Preliminary screening for antifungal activity

Out of fifty-five LAB strains (see Supplementary Table S1) screened against *F. oxysporum*, varying levels of inhibition were observed: three strains exhibited inhibition zones of 7–8 mm, two strains showed 9–11 mm, four strains showed 12–17 mm, and one showed > 18 mm (Fig. 1) after 5 days. An additional incubation was conducted on the selected ten strains with a zone of inhibition > 8 mm to assess stability and sustained inhibition. After 10 days, seven strains showed a significant reduction in inhibition (*p* < 0.01 or *p* < 0.001), while two (MYSVCF3 and MYSVCF5) maintained > 15 mm inhibition (Fig. 1). These strains, isolated from *Ficus racemosa* fruit, maintained stable inhibition zones > 15 mm, prompting further investigation into their combined probiotic and antifungal properties.

### Molecular identification

**Fig. 2:**
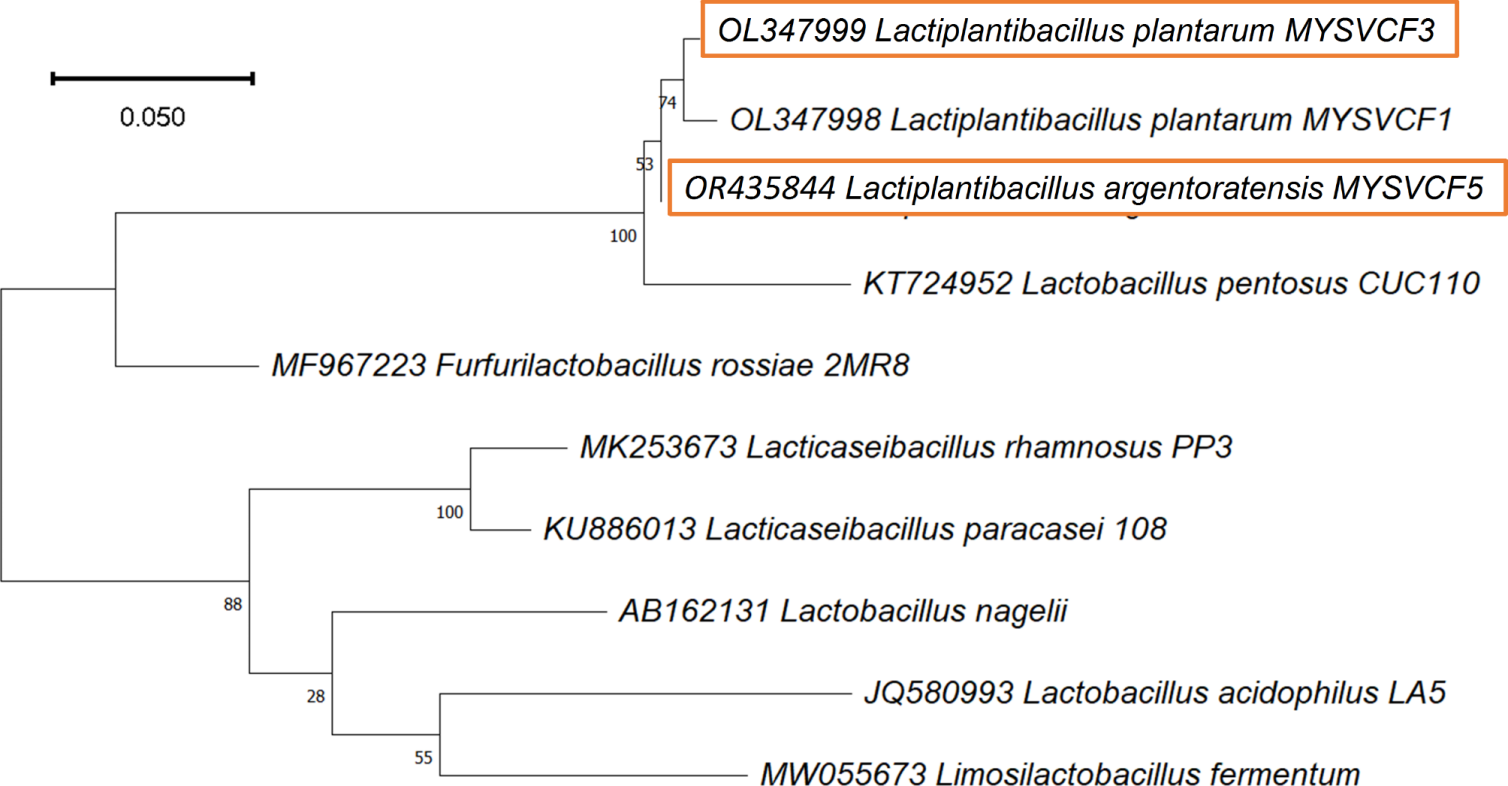
Phylogenetic tree showing the relative positions of LAB (MYSVCF3, MYSVCF5) obtained from fruits to that of reference strains.

The strains were identified after 16S rDNA sequencing as *Lactiplantibacillus* (*Lpb.*) *plantarum* MYSVCF3 and *Lpb. argentoratensis* MYSVCF5, with NCBI accession numbers **OL347999** and **OR435844**, respectively. Phylogenetic analysis revealed a high level of similarity with other known strains of *Lpb.* species (Fig. 2), particularly *Lpb. pentosus* suggesting they may have evolved from a common ancestor, suggesting genetic lineage and adaptation of these LAB strains.

### Probiotic attributes

#### Growth parameters

**Fig. 3:**
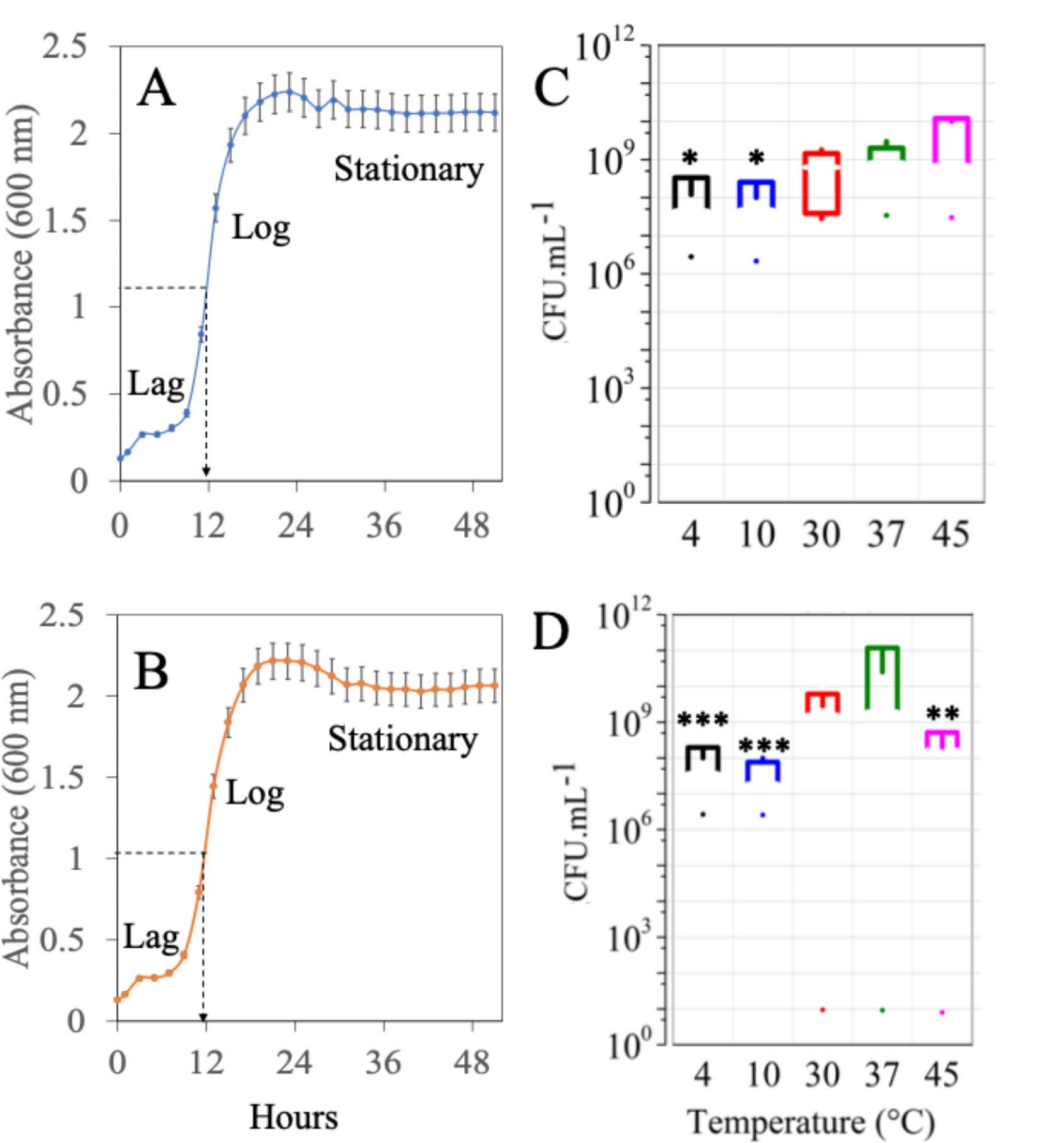
Growth curves of LAB (**A** and **B**) *Lpb. plantarum* MYSVCF3 (**A**, **C**) and *Lpb. argentoratensis* MYSVCF5 (**B**, **D**) at 37 °C. Viability and growth at different temperatures ranging from 4 to 45 °C (**C** and **D**) are shown. Statistical significance at *p ≤ 0.05; **p ≤ 0.01; ***p ≤ 0.001.

Both *Lpb*. *plantarum* and *Lpb*. *argentoratensis* were Gram-positive and catalase-negative, rod-shaped, and non-spore-forming bacteria. Except for D-arabinose for *Lpb*. *argentoratensis*, both could ferment all the sugars tested (Table 1). Optimal growth for *Lpb*. *plantarum* occurred at 37 °C (Fig. 3), reaching counts as high as 10^9^ CFU mL^−1^, whereas *Lpb*. *argentoratensis* thrived between 30 and 37 °C, with counts ranging from 10^9^ to 10^11^ CFU mL^−1^. However, temperatures above 37 °C or below 10 °C significantly reduced *Lpb*. *argentoratensis* growth by at least 2 logs (*p* < 0.01). Although a similar growth pattern was observed at 10 °C (*p* < 0.5), the temperature impact was less pronounced for *Lpb*. *argentoratensis* than *Lpb*. *plantarum*.

**Table 1:** The phenotypic traits and fermentation ability of the selected LAB isolates from tropical fruits.

Test	MYSVCF3	MYSVCF5
Gram character	+	+
Shape	rod	rod
Catalase	−	−
Hydrophobicity (%)	27 ± 2	42 ± 4
Carbohydrate fermentation		
Glucose	+	+
Lactose	+	+
Sucrose	+	+
Xylose	+	+
Maltose	+	+
D-Arabinose	+	-
Sorbitol	+	+
D-Raffinose	+	+

The strains *Lpb*. *plantarum* and *Lpb*. *argentoratensis* were characterized by rapid acidification during growth attributed to sugar fermentation and organic acid production. Despite initial lag phases of 7–8 h, both strains entered exponential growth lasting approximately 8 h before reaching the stationary phase (Fig. 3). Growth kinetics were similar, with division rates of 0.38–1.0 and 0.35 h^−1^, and generation times of 2.6 and 2.4 h, respectively. Maximum acidification (pH 4.5 ± 0.3) occurred between 10 and 17 h, aligning with the exponential growth phase characterized by specific growth rates of 0.24 and 0.26 h^−1^ at 37 °C for *Lpb*. *plantarum* and *Lpb*. *argentoratensis*, respectively. Both strains demonstrated sustained osmotic stress tolerance up to 7% NaCl concentration, with *Lpb*. *argentoratensis* showing decreased growth beyond this threshold.

#### Survival ability to gastrointestinal conditions

**Fig. 4:**
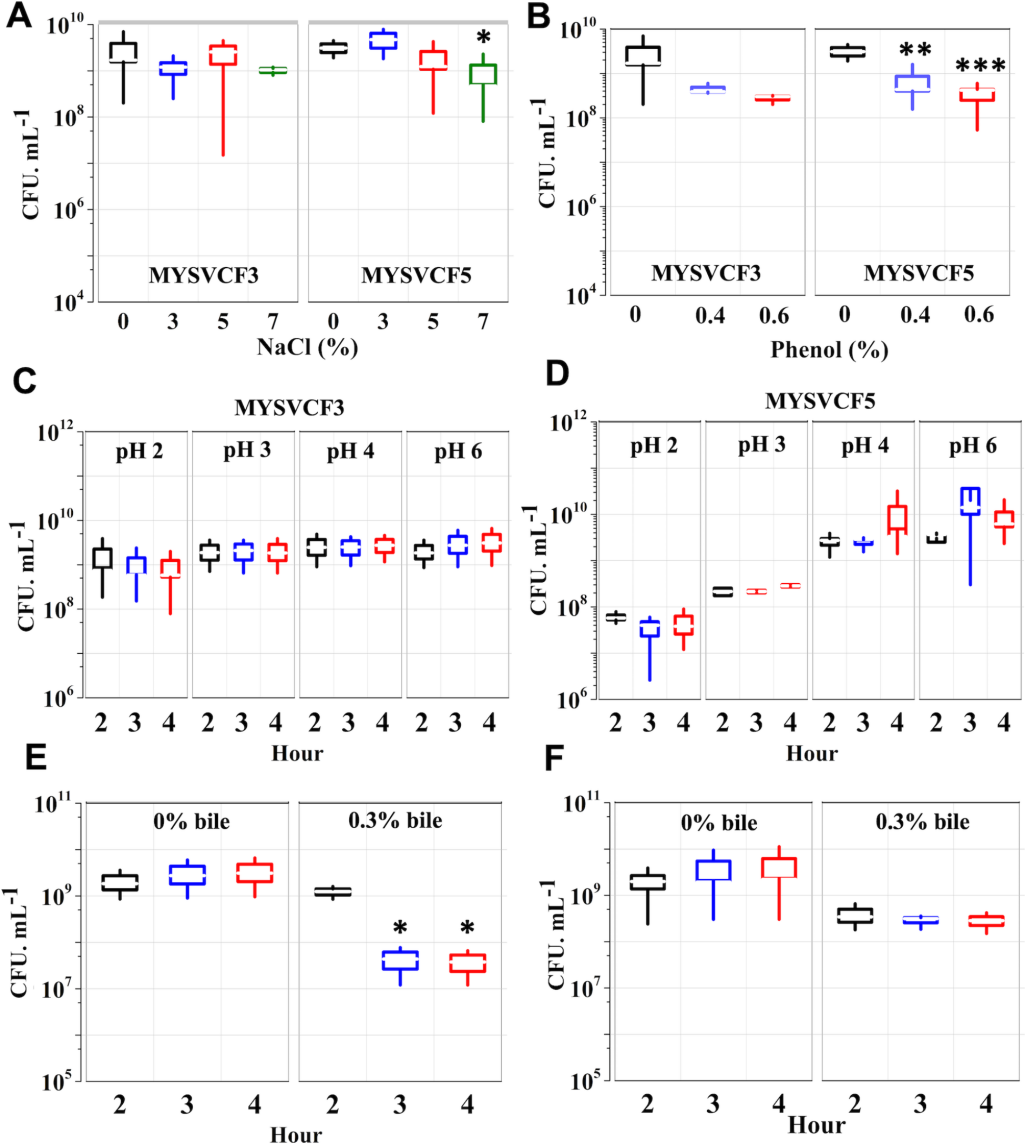
Tolerance of LAB at different conditions of osmotic stress (**A**), phenol (**B**), pH (**C**, **D**), and bile (**E**–**F**). The tolerance assay for *Lpb. plantarum* MYSVCF3 (A, B, C, and E) and *Lpb. argentoratensis* MYSVCF5 (**A**, **B**, **D**, **F**) are shown. Statistical significance at *p ≤ 0.05; **p ≤ 0.01; ***p ≤ 0.001.

Figure 4 illustrates the viability of LABs under simulated gastrointestinal conditions. Both *Lpb*. *plantarum* and *Lpb*. *argentoratensis* exhibited high tolerance to pH 2, with *Lpb*. *argentoratensis* maintaining > 95% and > 88% cell viability after 4 h (Fig. 4). Compared to the initial cell density (10^9^ CFU mL^−1^), a decrease in cell viability by a logCFU mL^−1^ after 4 h in pH 2 was observed, which is statistically insignificant (*p* > 0.05). In contrast, the viability of *Lpb*. *argentoratensis* declined significantly when phenol was added at concentrations greater than 0.4% (Fig. 4). while, even with 0.6% phenol, *Lpb*. *plantarum* demonstrated > 90% cell viability with 10^9^ CFU mL^−1^. Further, the strains exhibited tolerance to bile salts simulating the enteric phase (Fig. 4). Both *Lpb*. *plantarum*, and *Lpb*. *argentoratensis* showed excellent tolerance with *Lpb*. *argentoratensis* having better tolerance even after 4 h of exposure. The viability of *Lpb*. *plantarum* decreased by 15% (*p* < 0.05) after 3 h to the exposure to 0.3% bile (Fig. 4). Overall, the isolated LAB strains showed high tolerance to harsh simulated gastro-intestinal conditions making them ideal probiotic isolates.

#### Cell surface properties of LAB

The cell surface hydrophobicity of both isolates was evaluated, showing slightly higher hydrophobicity for *Lpb*. *argentoratensis* (42% ± 4) compared to *Lpb*. *plantarum* (27% ± 2) (Table 1). Over time, the percent aggregation increased, with strain *Lpb*. *argentoratensis* reaching 50% and *Lpb*. *plantarum* at 27% after 5 h (see Supplementary Fig. S1). By 24 h, *Lpb*. *argentoratensis* showed a marginal increase to 52% aggregation, while *Lpb*. *plantarum* increased to 57%.

#### Antibiotic susceptibility

Differences in antibiotic response were observed between *Lpb. plantarum* and *Lpb*. *argentoratensis* (Table 2). Both isolates were sensitive to ampicillin, clindamycin, erythromycin, streptomycin, and tetracycline, offering potential treatment options. While *Lpb*. *plantarum* was resistant to kanamycin, *Lpb*. *argentoratensis* exhibited resistance to chloramphenicol. Despite variations, the susceptibility of *Lpb*. *plantarum* and *Lpb*. *argentoratensis* to antibiotics did not differ significantly. Interestingly, both isolates exhibited resistance to vancomycin-specific antibiotics, suggesting inherent resistance mechanisms.

**Table 2:** Antibiotic susceptibility of selected LAB isolated from tropical fruits.

Antibiotics		Concentration (µg/disc)	LAB isolates*		Interpretive criteria
			MYSVCF3	MYSVCF5	
Ampicillin	Amp	10	S	S	≥ 16/ ≤ 12
Clindamycin	Cli	2	S	S	≥ 12/ ≤ 8
Chloramphenicol	Chl	30	S	R	≥ 18/ ≤ 13
Erythromycin	Ery	15	S	S	≥ 18/ ≤ 13
Kanamycin	Kan	30	R	S	≥ 18/ ≤ 13
Streptomycin	Ste	10	S	MS	≥ 15/ ≤ 11
Tetracycline	Tet	30	S	S	≥ 19/ ≤ 14
Vancomycin	Van	30	R	R	15

*R: resistance; S: sensitive; MS: moderately sensitive. The interpretation of sensitivity/resistance is expressed as the zone of inhibition (mm) of disc diffusion for potential *Lactobacillus* species (Charteris et al.^22^).

#### Antibacterial activity of LAB and its CFS

*Lpb. plantarum* and *Lpb*. *argentoratensis* exhibited comparable inhibition to the growth of selected pathogens such as *E. coli* (ATCC 25922)*, S. paratyphi* (ATCC 9150), and ESKAPE pathogens such as *P. aeruginosa* (ATCC 15422), and *S. aureus* (ATCC 6538) (see Supplementary Fig. S2). The CFS displayed antagonistic activity against all tested pathogens, with inhibition ranging from 70 to 80% *for Lpb*. *plantarum* and *Lpb*. *argentoratensis*. However, *S. aureus* and *P. aeruginosa* exhibited lower inhibition by *Lpb*. *argentoratensis* (74%) compared to other strains. Moreover, adding just 25% CFS was adequate to inhibit the pathogen growth completely, but the activity notably decreased after neutralization (nCFS) to 22–25% from the original 80% by both strains.

#### Hemolytic activity

*Lpb. argentoratensis* and *Lpb*. *plantarum* did not exhibit any red blood cell lysis in the hemolytic test. The clear yellow coloration surrounding the colony of *S. aureus* indicated that the organism had completely lysed red blood cells (*β*-hemolytic) (see Supplementary Fig. S3). Additionally, phenotypic tests for coagulase activity confirmed that both *Lpb. argentoratensis* and *Lpb*. *plantarum* are non-hemolytic and non-pathogenic.

### Antifungal activity of *Lpb. argentoratensis* MYSVCF5

#### Inhibition by bacterial cells of MYSVCF5

**Fig. 5:**
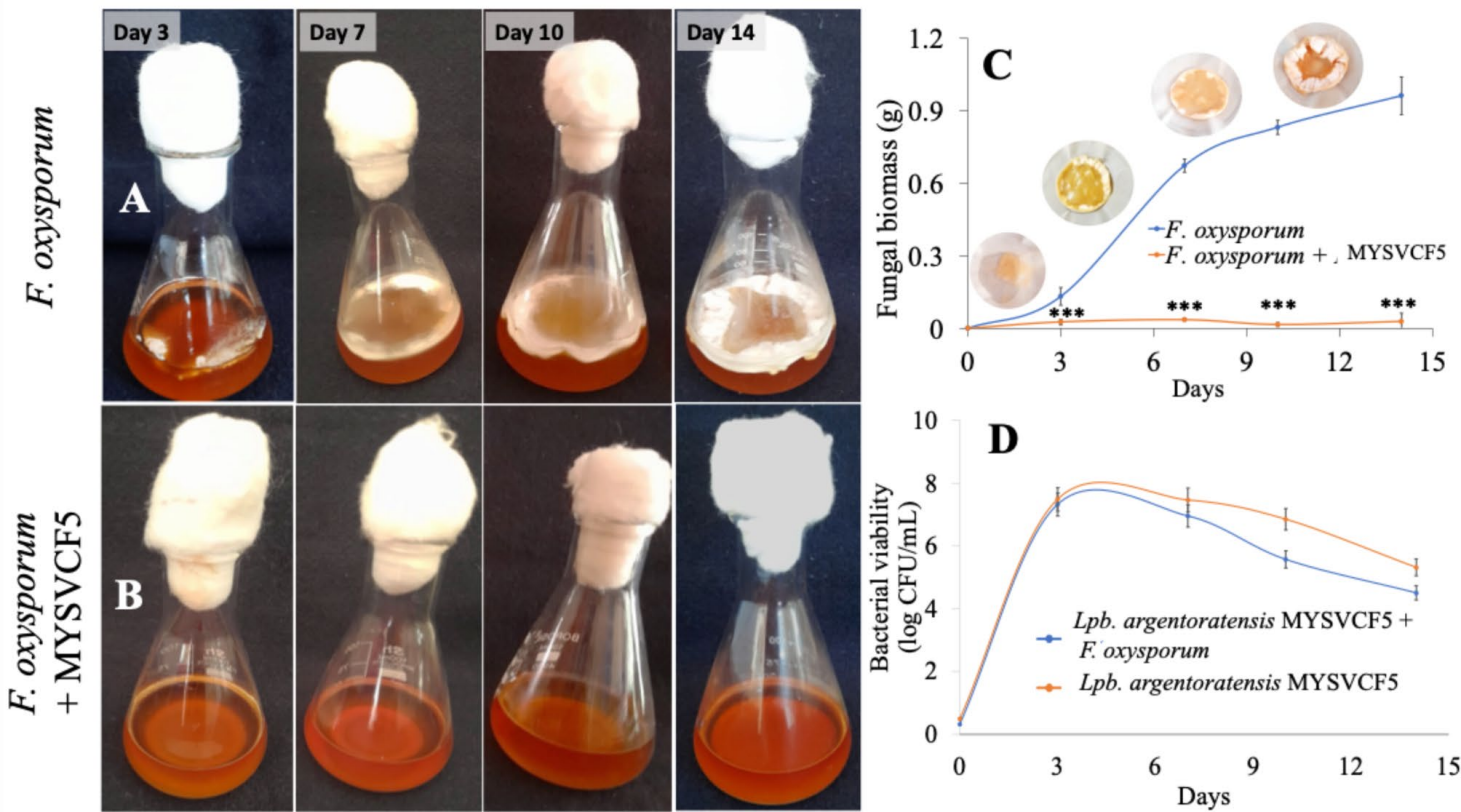
Time course growth of mycelial mat of *F. oxysporum* (control) (**A**, **C**) and *F. oxysporum* with cells of MYSVCF5 (test) (**B**, **D**). The growth of biomass of *F. oxysporum* under different conditions (**C**). The growth pattern of strain MYSVCF5 in the presence of *F. oxysporum* (**D**). Inset in C shows the fugal biomass obtained after filtration at different periods of the experiment. Statistical significance at *p ≤ 0.05; **p ≤ 0.01; ***p ≤ 0.001.

Figure 5A shows a steady growth of *F. oxysporum* in 14 days, characterized by a dense, white mat-like appearance. The biomass content progressively increased from < 0.01 g on day 1 to 0.9 g (by dry weight) on day 14 (Fig. 5B). In contrast, when viable cells of *Lpb*. *argentoratensis* were introduced, and complete inhibition (*p* < 0.01) of *F. oxysporum* growth was observed from day 1 onward (Fig. 5B). Meanwhile, *Lpb*. *argentoratensis* showed excellent viability throughout the incubation period, with an exponential phase lasting 3 days and a gradual decline as time progressed (Fig. 5D). These results indicate that *Lpb*. *argentoratensis* is effective in inhibiting the growth of *F. oxysporum*. The viability of the LAB cells was not affected by *F. oxysporum* (Fig. 5D).

#### Inhibition by CFS of MYSVCF5

**Fig. 6:**
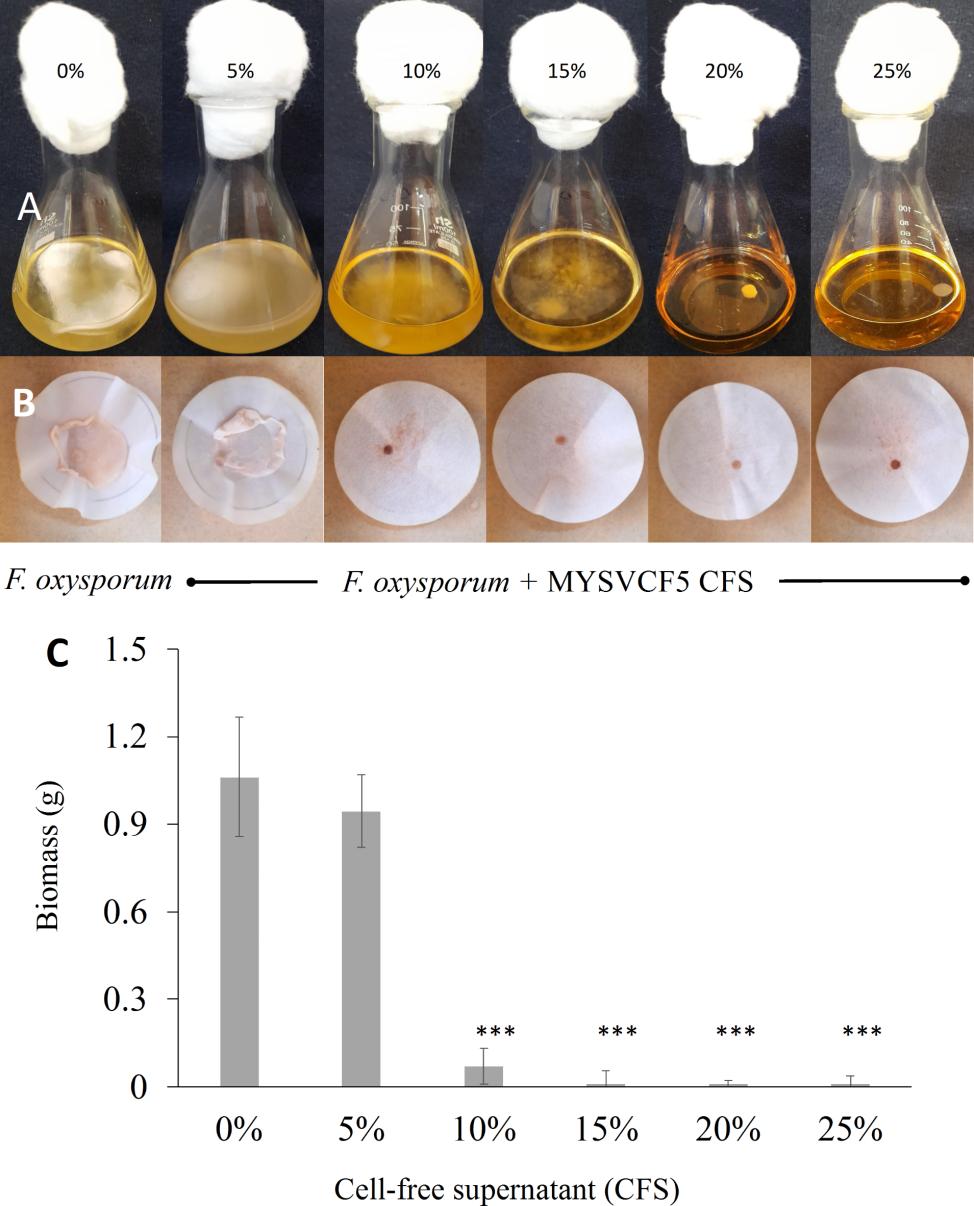
Antifungal activity as a function of inhibition of fungal biomass by cell-free supernatant (CFS) extracted from *Lpb. argentoratensis* MYSVCF5 (**A**–**C**). Experiments were performed with different concentrations (%) of CFS. Flasks showing growth of *F. oxysporum* with CFS of strain MYSVCF5 (**A**). Biomass yield obtained at different CFS treatments (**B** and **C**). Statistical significance at *p ≤ 0.05; **p ≤ 0.01; ***p ≤ 0.001.

Up to 5% of CFS, the inhibition of *F. oxysporum* was weak since the reduction of only 17% of the biomass was reached. However, by increasing the concentration to 10% CFS a significant reduction (*p* < 0.01) of 94% in fungal biomass yield was achieved, dropping from 1.1 to < 0.1 g (Fig. 6). Further increasing the CFS concentration to 20% led to a > 98% decrease in biomass yield (Fig. 6). Therefore, the inhibitory effects of CFS of *Lpb*. *argentoratensis* are considerably strong in inhibiting the growth of *F. oxysporum,* with a requirement of just 10% of crude extract. Moreover, the CFS retained its activity even after storage for 8 months at − 20 or 4 °C suggesting a strong and stable shelf-life (see supplementary Fig. S4).

#### The minimum fungicidal concentration of CFS

A CFS/media ratio of 5–10% was sufficient to completely inhibit the growth of *F. oxysporum*. At 0.6 mg/mL crude CFS, a 90% reduction was observed (see Supplementary Fig. S5). At this concentration, no visible growth of *F. oxysporum* was observed. An increase in CFS concentration increased activity and, as a result, an increase in inhibition. This clearly shows that 0.4–0.6 mg/mL CFS is effective at inhibiting *F. oxysporum* germination and growth, indicating CFS potential as a strong antifungal agent.

#### Conidial germination inhibition

**Fig. 7:**
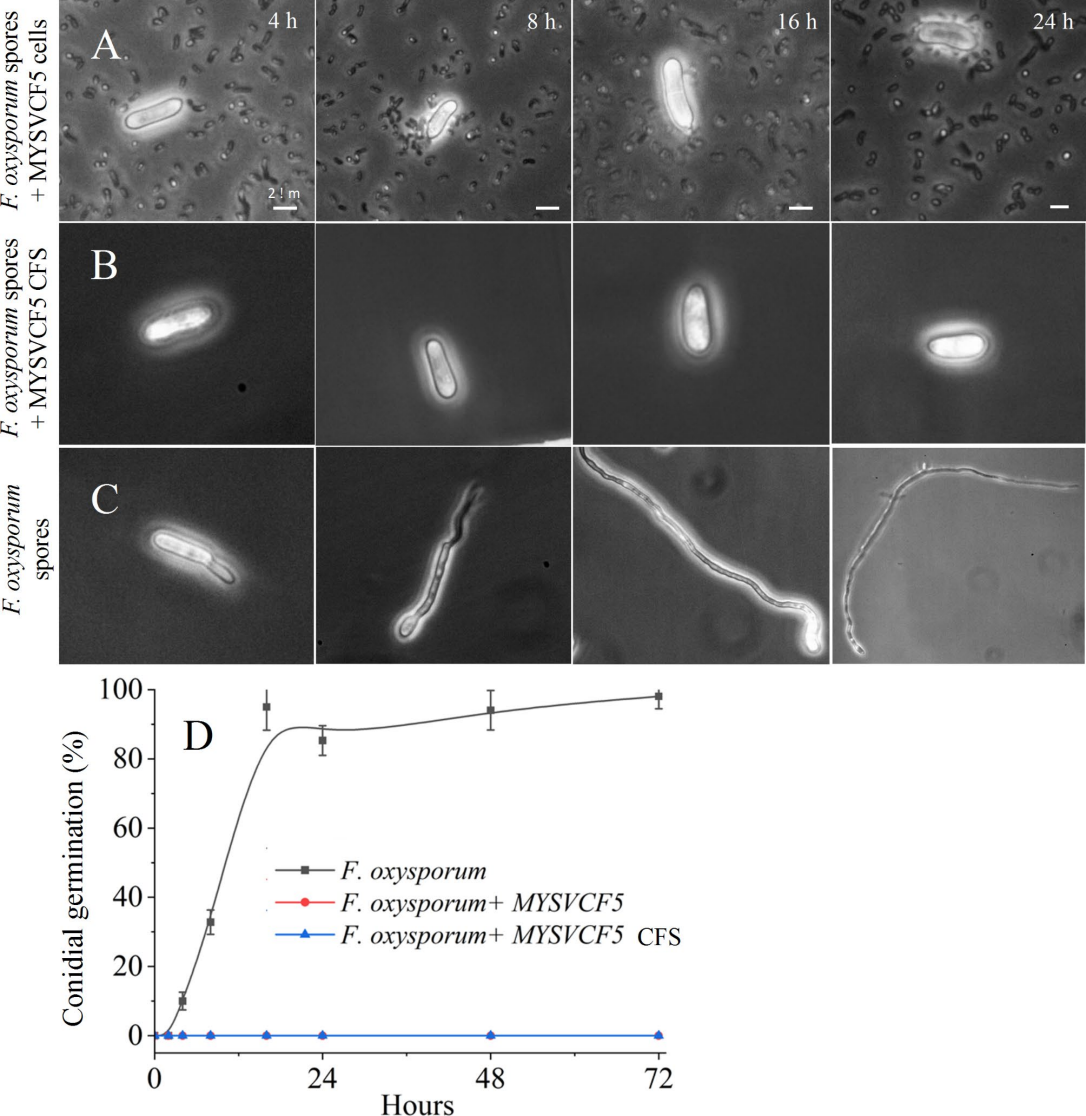
Microscopic observation of conidial germination inhibition *by Lpb. argentoratensis* MYSVCF5 cells (**A**), its CFS (**B**), and percent inhibition (**D**). Conidial germination in the absence of *Lpb. argentoratensis* MYSVCF5 cells or its CFS (**C**).

The presence of viable *Lpb*. *argentoratensis* cells significantly inhibited conidial germination and mycelial development (Fig. 7A). Conidial germination was impeded, with no germ tube formation observed even after 48 h in the presence of active LAB cells (Fig. 7A) or their CFS (Fig. 7B). In contrast, conidia germinated rapidly in the absence of LAB cells or CFS, with germ tube formation within 4 h and subsequent mycelial growth by 12 h, reaching 50% germination (10^6^ spores/mL) from apical cells (Fig. 7C and D). By 24 h, over 90% of the conidia had germinated with extensive mycelial growth (Fig. 7). The germination of conidia and formation of mycelia was much faster (< 24 h) when nutrient-rich, PDB medium was used.

#### Characterization of CFS

The CFS was subjected to heat, proteinase enzyme, and pH neutralization treatments to assess the stability and nature of its antifungal activity. The heat treatment preserved the inhibitory properties, but pH neutralization and proteinase K treatment resulted in the loss of antifungal activity allowing the fungi to sporulate and grow (see Supplementary Fig. S6). This indicates that the antifungal compound in the CFS is likely acidic, such as organic acids, and possibly contains heat-stable proteinaceous substances. However, the protein component of the CFS remains unidentified at this stage.

The chromatographic analysis of the CFS revealed a mixture of organic acids, with citric acid being the most abundant, constituting 67% of the total identified acids. This was followed by lactic acid at 16% and malic acid at 10%. The concentration of citric acid was 34.9 (± 0.3) µg/mL, and lactic acid 8.3 (± 0.003) µg/mL in the CFS (Table 3).

**Table 3:** Major organic acids composition of cell free supernatant (CFS) of strain *Lpb. argentoratensis* MYSVCF5.

Organic acids	Molecular weight (g mol^−1^)	CFS (µg mL^−1^)	% distribution
Citric acid	192.12	34.9 ± 0.3	66.9
Lactic acid	90.08	8.3 ± 0.003	16.5
Malic acid	116.1	5.2 ± 0.2	9.9
Succinic acid	118.09	1.7 ± 0.1	3.3
Pyruvic acid	88.06	1.3 ± 0.04	2.5

## Discussion

Microbial isolation from seven different fruits revealed LAB counts ranging from 9 to 10 log CFU/g, with notably high counts in *Solanum nigrum* and *Annona muricata*. The abundance of viable LABs was comparable to previous studies^17^. Pineapple, papaya, grapes, blackberries, and kiwis showed counts ranging from 10^2^ to 10^6^ CFU/g, while grape and Musa had lower counts (< 10^2^ CFU/g)^30^. Fifty-five LAB strains were obtained after several screening stages, differing from previous reports. LAB species varied among fruits, with *Lactobacilli* predominating^31^. Previous studies have reported *L. plantarum*, *Weisella cibaria*, *Weisella paramesenteroides*, *Leuconostoc mesenteroides*, *Leuconostoc citreum* in ripe mulberries, watermelons, peaches, and coffee cherries. Yet, *L. plantarum, L. brevis L. fermentum* and *L. paracasei* are the most common species of *Lactobacillus* isolated from fruits^31,32^. Of the fifty-five isolates, only eleven strains showed inhibition to the growth of *F. oxysporum* (Fig. 1). Among these strains *Lpb. plantarum* and *Lpb. argentoratensis* exhibited the highest inhibition (Fig. 1). Therefore these two strains were selected for further analysis. Both selected strains were identified as hydrophobic, catalase-negative Gram-positive rods, likely belonging to the *Lactiplantibacillus* or *Lactobacillus* genus. 16S rDNA analysis revealed a close relationship to *Lpb. plantarum* and *Lpb. argentoratensis*, both known for their probiotic properties. Despite their taxonomic distinction, these strains exhibited high homology and potential as beneficial microorganisms.

Both *Lpb. plantarum* and *Lpb. argentoratensis* displayed sensitivity to common antibiotics, suggesting safe and effective use in probiotic formulations. They also demonstrated resistance to vancomycin-specific antibiotics, indicating potential resistance mechanisms or unique cell wall characteristics^31^. In vitro assays confirmed their tolerance to acidic conditions and bile contents typical of the gastrointestinal (GI) tract, enhancing their ability to colonize the host’s gut. The GI environment, known for its acidity and bile presence, poses challenges to bacterial viability. Both strains survived pH 2 and tolerated human bile up to 0.5%, with *plantarum* exhibiting slightly better tolerance. Actual stomach pH variations, buffered by meals and gastric juices, may offer protection against bacterial viability. Therefore, the resilience of probiotic strains depends on inherent resistance and the food matrix.

In screening for antimicrobial activity, the strains exhibited both antibacterial and antifungal properties. Their antibacterial effects were observed against various pathogenic and indicator bacteria, including *S. aureus, P. aeruginosa, S. paratyphi*, and *E. coli*, albeit with varying potency. Interestingly, both LAB cells and CFS revealed a positive inhibition of these pathogens. One of the functional attributes of probiotics lies in the ability to produce antimicrobial compounds, encompassing a combination of organic acids, short-chain fatty acids, and other bacteriocin toxins^31^. *L. plantarum* and *P. pentosaceus* have shown significant antibacterial properties against a variety of pathogens, including *S. aureus* and *E. coli*, in multiple studies^20,32,33^. Additionally, strains like *L. plantarum* and *Lacticaseibacillus. rhamnosus* has been shown to inhibit *P. aeruginosa*^9,20^* and S. pyogenes*^34^, suggesting a commonality in the antibacterial profiles of LAB strains, including those isolated in the current study. However, *S. typhi* is less frequently studied, and while some studies report antimicrobial effects against enteric pathogens, it is not always the focal point^35^.

Nevertheless, previous studies indicate that LAB strains can exhibit activity against enteric pathogens. The synthesis of these antimicrobial metabolites by LAB bears potential benefits not only for food preservation but also for mitigating the proliferation of pathogens^36^.

The proliferation of *Fusarium* sp. is influenced by environmental factors such as agricultural practices and storage conditions, including temperature and moisture content. LAB is an important commensal microorganism with documented antifungal attributes and bio-preservation capabilities^21,37,38^. Among LABs, *Lactobacillus* species, particularly *L. plantarum*, are known for effectively suppressing *Fusarium* growth^39^. While *plantarum* is recognized for inhibiting *Aspergillus, Listeria monocytogenes*, etc., the antifungal activity of *Lpb. argentoratensis* isolated from tropical fruits is yet to be studied but has great potential. The microconidia germination ceased completely in the presence of cells or CFS of *Lpb. argentoratensis*. A minimal fungicidal concentration of 1 mg/mL of CFS could effectively inhibit both mycelial and conidial germination of *F. oxysporum*.

Given that the LABs are known to produce organic acids^38,40^, it was anticipated that the organic acids were primarily responsible for the observed antifungal effect, as pH may have a significant impact as an antifungal agent. Organic acids produced by LABs can disrupt microbial metabolic activity by penetrating cell membranes in their undissociated form, lowering intracellular pH. While previous studies have identified organic acids like acetic acid and propionic acid in *plantarum*^40^, similar investigations on *Lpb. argentoratensis* are lacking. Thus, this study is the first evidence of the antifungal activity of *Lpb. argentoratensis*. The CFS after neutralization to pH 7 or treatment with proteinase K resulted in the loss of inhibition. However, sustained inhibition was observed after heat treatment. Thus, it was inferred that the antifungal metabolite produced by *Lpb. argentoratensis* comprised both organic acids and thermally stable proteinaceous substances. Subsequent analysis of the organic acid profile confirmed the presence of several known organic acids. Citric acid and lactic acid together accounted for 82% of the organic acid pool. Citric acid, in particular, was identified as the most relevant antifungal compound, as it accumulated to concentrations matching the minimum fungicidal concentration against fungal spores. Although organic acids likely contribute to this activity, the presence of any protein component in the cell-free supernatant has yet to be confirmed. Additional studies are needed to better comprehend the mechanisms through which *Lpb. argentoratensis* inhibits *F. oxysporum*.

The antifungal properties of LAB have been widely recognized, particularly those derived from various natural sources, such as fermented foods and fruits. Previous studies have reported a range of LAB strains, including *Lactobacillus plantarum* and *Pediococcus pentosaceus*, demonstrating activity against several fungal species like *F. oxysporum*, *Penicillium nordicum*, and *Colletotrichum.* Organic acids are recognized as primary antifungal agents, with their efficacy often enhanced by synergistic metabolites like proteins and secondary compounds^40^. Benzoic acid, cinnamic acid, and their derivatives have been shown to exhibit antifungal activity. Moreover, phenolic compounds like caffeic acid, vanillic acid, and vanillin further implicate the diversity of antifungal metabolites^40^. Proteinaceous inhibitors, although implicated, remain inadequately characterized in terms of their structural and functional properties^41^. Few studies indicated that a combination of organic acids and volatile organic compounds, such as 2-Nonanone and 2-Undecanone, contribute to antifungal activity against *Fusarium* species^40^. Despite these insights, the lack of a direct correlation between metabolic profiles and antifungal efficacy still limits comprehensive understanding. Nonetheless, this study introduces a novel LAB strain derived from *Solanum nigrum*, which exhibits exceptional antifungal activity against *F. oxysporum*, marking a unique addition to the pool of LAB strains.

## Implications

The effective management of fungal plant diseases caused by *Fusarium* species, including *F. oxysporum* has presented a persistent challenge since its adverse impact on agricultural yields was recognized. Environmental factors such as cool, damp weather, particularly during growth necessitate alternative, economic, and environmentally sustainable solutions. The use of LAB as probiotics and antifungal agents offers a promising alternative. LABs possess several advantages, including economic feasibility, safety, ease of production, and dual roles as probiotics and biocontrol agents. These attributes make them attractive for integration into crop protection strategies.

The diverse LAB population found in fruits is a valuable resource for developing probiotic-based solutions. However, the specific proteinaceous compound (if any) responsible for antifungal activity was not identified in this study. Additionally, variability in LAB strain effectiveness due to environmental factors or host fruit origin may affect the efficacy of the strain. The practical application of LABs in agricultural settings also requires further exploration to address challenges related to scalability and delivery. Therefore, future research should prioritize the characterization of CFS to elucidate specific antifungal mechanisms and identify bioactive compounds. In vivo studies are essential to evaluate the real-world efficacy of LAB strains against plant pathogens under diverse agricultural conditions. Optimizing fermentation parameters to enhance LAB growth and boost the production of antifungal metabolites can further strengthen their role in sustainable agricultural practices.

## Conclusion

In this study, multiple LAB strains were isolated from various tropical fruits. The highest counts were observed in *Solanum nigrum*. Among the isolates, *Lactiplantibacillus plantarum* MYSVCF3 and *Lpb. argentoratensis* MYSVCF5, derived from cluster fig, exhibited stable probiotic properties in vitro. Both strains demonstrated antibacterial and antifungal activity against *Fusarium oxysporum*, with *Lpb. argentoratensis* showing stable and consistent inhibition. The antifungal activity was likely mediated by organic acids, with citric acid at 34.9 µg/mL identified as the most abundant compound produced by *Lpb. argentoratensis*. This study enhances the knowledge of LAB diversity in selected tropical fruits. The use of *Lpb. argentoratensis* as a biocontrol agent offers ecological benefits by reducing reliance on synthetic fungicides. Thus lowering the environmental pollution and mitigating adverse effects on non-target organisms.

## Electronic supplementary material

Below is the link to the electronic supplementary material.

Supplementary Material 1

## Data Availability

All data generated or analyzed during this study are included in this published article (and its Supplementary Information files). The sequences of bacterial isolates from this study can be found in the NCBI GenBank repository under NCBI accession numbers **OL347999** (https://www.ncbi.nlm.nih.gov/nuccore/OL347999.1/) and **OR435844** (https://www.ncbi.nlm.nih.gov/nuccore/OR435844)
