# Supplementary material for: Tropical fruit-derived Lactiplantibacillus as potential probiotic and antifungal agents against Fusarium oxysporum
Source: Sci Rep. 2025 Jan 16;15:2144. doi: 10.1038/s41598-025-85190-0 (PMC11739408; doi:10.1038/s41598-025-85190-0)
Supplement: Supplementary file 1 — Supplementary Material 1 [file 41598_2025_85190_MOESM1_ESM.docx]

**Electronic supplementary information**

**Tropical fruit-derived *Lactiplantibacillus* as potential probiotic and antifungal agents against *Fusarium oxysporum***

**R. Vasundaradevi^a^, M. Sarvajith^a,1^, S. Divyashree^a^, N. Deepa^a^, Premila N. Achar^b*^, M. Y. Sreenivasa^a^***

^a^Molecular Mycotoxicology Lab, Department of Studies in Microbiology, University of Mysore, Manasagangotri, Mysuru-570 006, India

^b^Department of Molecular and Cellular Biology, Kennesaw State University, Kennesaw, GA30144, USA

*Correspondence:

**Dr. Premila N. Achar**

Professor,

Department of Molecular and Cellular Biology, Kennesaw State University,

Kennesaw, GA30144, USA

E-mail id: [pachar@kennesaw.edu](mailto:Pachar@kennesaw.edu)

*Correspondence:

**Dr. M. Y. Sreenivasa**

Professor and Head,

Department of Studies in Microbiology, University of Mysore,

Mysuru-570 006, India.

E-mail id: [sreenivasamy@gmail.com](mailto:sreenivasamy@gmail.com), [mys@microbiology.uni-mysore.ac.in](mailto:mys@microbiology.uni-mysore.ac.in)

^1^Present address:

WDRC, Biological and Environmental Science and Engineering Division,

King Abdullah University of Science and Technology,

Thuwal 23955-6900, Kingdom of Saudi Arabia

E-mail id: [sarvajith.manjunath@kaust.edu.sa](mailto:sarvajith.manjunath@kaust.edu.sa)

No. of pages: 5

No. of figures: 4

**List of figures and tables**

Figure S1. Time course increase in aggregation index of *Lpb. plantarum* MYSVCF3 and *Lpb. argentoratensis* MYSVCF5 isolated from fruit.

Fig. S2 Antibacterial activity of *Lpb. plantarum* MYSVCF3 (A) and *Lpb. argentoratensis* MYSVCF5 (B) against selected pathogens and ESKAPE pathogens. For this, viable LAB cells, crude cell-free supernatant (CFS) and neutralized CFS was used. Statistically significant variation in relation to control at **p≤0.05*; ** *p≤0.01; ***p≤0.001*.

Figure S3. Hemolytic activities of *Staphylococcus aureus* showing β -hemolysis (A) and *Lpb. argentoratensis* MYSVCF5 showing no lysis on blood agar media.

Figure S4. Antifungal activity of cell-free supernatant (CFS) of *Lpb. argentoratensis* MYSVCF5 against *F. oxysporum*. The activity was tested using the stored CFS for over 8 months at 4 °C (A) and -20 °C (B). 0, 10, 20, and 30% represent the percentage of stored CFS used.

Figure. S5 Minimum fungicidal inhibitory concentration of crude cell-free supernatant (CFS) extracted from *Lpb. argentoratensis* MYSVCF5. Statistically significant variation in relation to control at **p≤0.05*; ** *p≤0.01; ***p≤0.001*.

Figure S6. Residual antifungal activity after treatment of cell-free supernatant (CFS) of *Lpb. argentoratensis* MYSVCF5 against *F. oxysporum*. Treatment conditions: pH neutralization, proteinase K, and heat treatment at 80 ℃ are shown.

Table S1. Details of the selected tropical fruits used in this study to isolate lactic acid bacteria.

Table S1. Details of the selected tropical fruits used in this study to isolate lactic acid bacteria.

| Scientific name | Common name | Collection season | Month and year of collection | No. of pin-point colonies |
| --- | --- | --- | --- | --- |
| *Tinospora cordifolia* | Amrutha balli | Spring | February 2020 | 8 |
| *Ficus benghalensis* | Banyan tree | Winter | January 2020 | 8 |
| *Solanum nigrum* | Black night shade | Spring | February 2020 | 14 |
| *Couroupita guianenis* | Cannon ball tree | Summer | June 2019 | 4 |
| *Ficus racemosa* | Cluster fig | Winter | January 2020 | 10 |
| *Musa* | Nanjangudu rasabale | Spring | February 2020 | 4 |
| *Annona muricata* | Soursop | Spring | February 2021 | 7 |

Figure S1. Time course increase in aggregation index of *Lpb. plantarum* MYSVCF3 and *Lpb. argentoratensis* MYSVCF5 isolated from fruits.

Fig. S2 Antibacterial activity of *Lpb. plantarum* MYSVCF3 (A) and *Lpb. argentoratensis* MYSVCF5 (B) against selected pathogens and ESKAPE pathogens. For this, viable LAB cells, crude cell-free supernatant (CFS) and neutralized CFS (nCFS) was used. Statistically significant variation in relation to control at **p≤0.05*; ** *p≤0.01; ***p≤0.001*.

Figure S3. Hemolytic activities of *Staphylococcus aureus* showing β -hemolysis (A), *Lpb. plantarum* MYSVCF3 (B), and *Lpb. argentoratensis* MYSVCF5 (C) showing no lysis on blood agar media.

Figure S4. Antifungal activity of cell-free supernatant (CFS) of *Lpb. argentoratensis* MYSVCF5 against *F. oxysporum*. The activity was tested using the stored CFS for over 8 months at 4 °C (A) and -20 °C (B). 0, 10, 20, and 30% represent the percentage of stored CFS used.

Figure. S5 Minimum fungicidal inhibitory concentration of crude cell-free supernatant (CFS) extracted from *Lpb. argentoratensis* MYSVCF5. Statistically significant variation in relation to control at **p≤0.05*; ** *p≤0.01; ***p≤0.001*.

Figure S6. Residual antifungal activity after treatment of cell-free supernatant (CFS) of *Lpb. argentoratensis* MYSVCF5 against *F. oxysporum*. Treatment conditions: pH neutralization, proteinase K, and heat treatment at 80 ℃ are shown.
